# Protective Role of Multiple Essential Minerals Against Cadmium-Related Cognitive Decline in Middle-Aged and Older Adults: A Prospective Study

**DOI:** 10.3390/nu17182910

**Published:** 2025-09-09

**Authors:** Jing Yang, Zongyao Li, Yongbin Zhao, Yanzhen Hu, Xinyang Guo, Xi Kang, Zhenyu Wu, Chang Su, Tao Zhang

**Affiliations:** 1 Chinese Center for Disease Control and Prevention, Beijing 102206, China; 2 Department of Biostatistics, School of Public Health, Cheeloo College of Medicine, Shandong University, Jinan 250012, China; 3 Institute for Medical Dataology, Shandong University, Jinan 250002, China; 4 Department of Epidemiology and Statistics, School of Public Health, Tianjin Medical University, Tianjin 300070, China; 5 Tianjin Key Laboratory of Environment, Nutrition and Public Health, Tianjin 300070, China; 6 Key Laboratory of Prevention and Control of Major Diseases in the Population, Ministry of Education, Tianjin Medical University, Tianjin 300070, China; 7 National Institute for Nutrition and Health, Chinese Center for Disease Control and Prevention, Beijing 100050, China; 8 Key Laboratory of Public Nutrition and Health, National Health Commission of the People’s Republic of China, Beijing 100050, China; 9 School of Public Health, Fudan University, Shanghai 200032, China

**Keywords:** cadmium exposure, essential minerals, cognitive function, effect modification

## Abstract

**Background:** Cadmium (Cd) exposure is linked to cognitive decline in middle-aged and older adults, but the modifying role of essential minerals is unclear. This study aimed to identify key protective minerals and quantify their joint antagonistic effect against Cd neurotoxicity. **Methods:** Baseline serum minerals and urinary Cd were measured in 6795 adults (≥40 years) from the 2015 China Health and Nutrition Survey. Cognitive function (MMSE) was assessed after 3 years. Associations were analyzed using multiple linear regression and Quantile g-computation (QGC) for joint effects. Combined exposure groups and interaction terms were assessed. Restricted cubic spline (RCS) models explored potential nonlinear dose–response relationships. **Results:** Participants in the highest urinary Cd quartile had significantly lower MMSE score (β = −0.09, 95% CI: −0.15, −0.02) than the lowest quartile. Serum calcium (Ca), ferrum (Fe), magnesium (Mg), selenium (Se), and phosphorus (P) were positively associated with MMSE. QGC revealed that the joint effect of Cd and the 5-mineral mixture (β = 0.10, 95% CI: 0.05, 0.14) was weaker than the protective effect of the 5-mineral mixture. Any high-mineral group had significantly higher MMSE score compared to the high-Cd/low-mineral group. **Conclusions:** Essential minerals Ca, Fe, Mg, Se, and P effectively antagonize Cd-associated cognitive decline. Their combined exposure demonstrates significant protective effects, providing key evidence for precision nutrition and environmental health risk management in Cd-exposed populations.

## 1. Introduction

Cognitive decline, a major risk factor for mild cognitive impairment (MCI) and dementia, significantly impacts independence and quality of life in aging populations [1,2]. Rapid population aging has transformed this into a substantial societal challenge, particularly severe in China where 14.2% of the population was ≥65 years in 2021 [3]. With an estimated 16.99 million Chinese individuals affected by Alzheimer’s disease and related dementias in 2021, representing 23.6% of the global burden, the social and economic costs are escalating [4,5]. Cognitive decline results from complex interactions between genetic, environmental, and lifestyle factors, including exposure to neurotoxic chemicals such as cadmium (Cd) [4]. Beyond endogenous risks such as age, genetics, and medical history, the contribution of exogenous factors like environmental pollutant exposure warrants significant attention [6]. A growing body of evidence specifically links cadmium exposure to neurodegenerative pathologies including Alzheimer’s disease. Numerous studies have consistently reported elevated cadmium levels in the blood and urine of individuals with Alzheimer’s disease compared to healthy controls, implicating a potential role for cadmium in the progression of the disease [7,8]. Furthermore, ecological and epidemiological investigations have indicated correlations between environmental pollution levels, with particular emphasis on heavy metal contamination, and increased incidence and prevalence of Alzheimer’s disease, highlighting the public health significance of environmental neurotoxicants [9]. Cd bioaccumulates in humans primarily through tobacco smoke, contaminated water, and crops, with a biological half-life of 20–30 years [10]. Cd can penetrate the blood–brain barrier, inducing neurotoxicity through mechanisms including oxidative stress, mitochondrial dysfunction, neuroinflammation, and accelerated neuronal apoptosis [11]. Epidemiological evidence increasingly links elevated Cd levels to cognitive impairment in middle-aged and older adults [12,13,14].

While cadmium (Cd) exposure is an established environmental risk for cognitive decline, emerging evidence suggests dietary intake of essential minerals may modify its neurotoxic effects. Minerals such as calcium (Ca), iron (Fe), magnesium (Mg), selenium (Se), and phosphorus (P) are critical for neural signaling, synaptic plasticity, and antioxidant defenses—biological processes frequently disrupted by Cd [15,16,17,18,19]. Understanding their potential to counteract Cd-induced neurotoxicity could inform nutritional interventions for cognitive preservation. Epidemiological studies suggest positive associations between serum selenium and phosphorus levels and cognitive function [20,21]. Furthermore, previous studies indicate that magnesium, calcium, and selenium confer neuroprotection by mitigating oxidative stress and inflammation, supporting blood–brain barrier integrity, and maintaining myelin and axonal structure [22,23]. Iron is also required for the normal development of cognitive function [16]. However, critical knowledge gaps persist regarding the modulatory role of essential minerals in Cd-related cognitive decline [24]. First, population-level evidence on effect modification between Cd exposure and essential minerals remains limited [12]. Second, prior studies focus predominantly on single-mineral analyses, overlooking potential synergistic or antagonistic interactions among multiple minerals [25]. Third, existing research has centered on specialized cohorts (e.g., pregnant women), leaving heterogeneity across broader demographic subgroups unexplored [26].

This study leverages nationally representative data from the China Health and Nutrition Survey (CHNS) prospective cohort to examine the association between baseline cadmium exposure and serum minerals (measured in 2015) and subsequent cognitive function (assessed in 2018). Urinary cadmium and serum mineral concentrations served as prior exposures, with cognitive decline evaluated three years later using the Mini-Mental State Examination (MMSE). Our objectives are threefold: First, to identify key essential minerals potentially capable of modulating cadmium-associated cognitive impairment; second, to quantify the combined antagonistic effect of multiple mineral exposures on cadmium-related neurotoxicity; and third, to analyze effect heterogeneity across demographic subgroups. Ultimately, this research aims to provide a scientific foundation for developing precision nutrition interventions targeting cognitive health in the context of environmental Cd exposure.

## 2. Materials and Methods

### 2.1. Study Population

**Figure 1:**
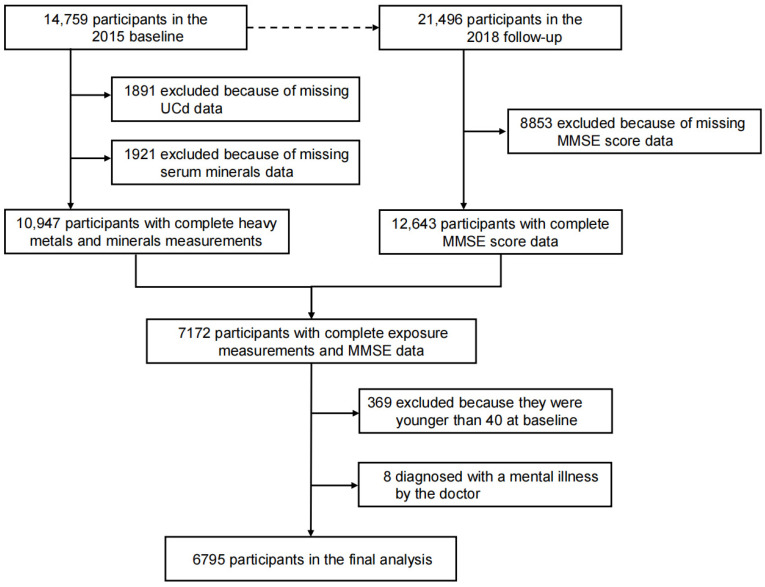
Workflow of participants included in the analyses. Abbreviations: UCd, urinary cadmium; MMSE, mini-mental state examination.

This study was based on data from the CHNS, an ongoing prospective cohort that began in 1989 with follow-up every 2–4 years. It employed multi-stage random cluster sampling to collect longitudinal data on sociodemographic factors, dietary patterns, physical activity, health status, and behavioral changes at both household and individual levels [27,28]. The survey covers a diverse range of 15 provinces and municipalities, with survey sites selected through a stratified sampling process by geographic location and economic indicators. This approach was designed to ensure a balanced representation of urban and rural areas across different socioeconomic strata in the sample [27]. Based on the availability of data on serum concentrations of essential minerals and urinary Cd (UCd), the 2015 cycle of CHNS (n = 10,947) was included in this analysis. We excluded participants under 40 years old, those missing samples for any of the 5 serum essential minerals (calcium [Ca], ferrum [Fe], magnesium [Mg], selenium [Se], phosphorus [P]) and UCd measurements, and those without sufficient information to evaluate cognitive function. Finally, a total of 6795 participants aged ≥40 years from the 2015 baseline wave were included in the study (Figure 1). All participants who enrolled in the study signed a written informed consent to participate in the CHNS. The study received institutional review and approval from the Ethics Committee of the Institute of Nutrition and Health of the Chinese Center for Disease Control and Prevention (No. 201524).

### 2.2. Laboratory Quantification of UCd and Serum Minerals

Participants had fasted for 8 to 12 h at the time of blood sample collection. Standard operating procedures regarding blood-sample collection, processing, and storage were followed. Methods for measurement and quality control of serum samples were detailed in previous studies [28]. The UCd concentration was measured using inductively coupled plasma-Mass Spectrometry (ICP-MS). The ICP-MS analytical methodology is detailed in Supplementary Method S1. Serum concentrations of five serum essential minerals were quantified using standardized analytical platforms. Serum concentrations of Ca, Fe, Mg, and P were measured by automated colorimetric assays on a Roche Cobas 8000 C701/702 automatic biochemical analyzer (Roche Diagnostics, Indianapolis, IN, USA). Ca was quantified via the colorimetric assay method. Fe was analyzed using the ferrozine method. P was assessed by the phosphomolybdate UV method, and Mg was measured via a chromogenic complex indicator method. Serum Se concentration was determined by high-resolution inductively coupled plasma mass spectrometry (ICP-MS; Agilent 7700 series). All measurements adhered to strict quality control protocols, including daily calibration with Roche c.f.a.s calibration reagents, internal quality control using Bio-Rad Liquichek^TM^ General Chemistry Controls Level 1 & 2, and external validation through the National Routine Chemistry A Program (China MOH). Concentrations below the lower limit of quantitation (LLOQ) were replaced by LLOQ divided by the square root of 2. Concentrations are reported as: Ca (mmol/L), Mg (mmol/L), Fe (μmol/L), P (mmol/L), Se (μg/L), and UCd (μg/L). The LLOQ, detection rate, and distribution of serum concentrations for the 5 serum essential minerals and UCd are shown in Table S1.

### 2.3. Cognitive Function Assessment

Cognitive function was assessed on a single occasion using the Mini-Mental State Examination (MMSE), at the conclusion of the 3-year follow-up in 2018. The MMSE evaluated five domains: orientation (10 points; assessing knowledge of time and place), registration (3 points; immediate recall of three words), attention/calculation (5 points; working memory and serial subtraction), recall (3 points; delayed recall of the three previously registered words), and language/praxis (9 points; including naming, repetition, comprehension, reading, writing, and figure copying). Trained interviewers administered the validated Chinese version in controlled environments (noise < 50 dB), with raw score (0–30) recorded [29,30]. A higher total MMSE score indicates better cognitive function. Continuous score was used in regression analyses and other models.

### 2.4. Anthropometric and Laboratory Measurements

The body weight and height of all participants were measured by trained examiners using calibrated equipment following standardized procedures. After participants rested for at least five minutes, their sitting blood pressure was measured three times using a mercury sphygmomanometer. The mean values of three measurements of systolic and diastolic blood pressure were used for analysis.

Fasting blood glucose (FBG) was measured using the hexokinase method on a cobas c701/c702 automated biochemical analyzer (Roche Diagnostics, Indianapolis, IN, USA) with a glucose detection kit. Glycated hemoglobin A1c (HbA1c) levels were assessed using a high-performance liquid chromatography system (Tosoh Corporation, Tokyo, Japan) [31]. Urine creatinine (UCr) concentration was quantified enzymatically on the same Roche Cobas 8000 C701/702 analyzer. All samples adhered to strict quality control at the National Center Laboratory in Beijing, China.

### 2.5. Covariates

The CHNS obtained personal information, including sociodemographic information (age, sex, residence, education level, annual household income), dietary intake (total energy intake, total fat intake), and lifestyle (smoking, alcohol consumption, physical activity) by self-reporting in the questionnaire in each survey year. Dietary intake, including total energy and total fat, was assessed based on the average daily intake derived from three consecutive 24 h dietary recalls during the 2015 survey wave, with nutrient calculations performed according to the Chinese food composition table. Physical activity was measured in metabolic equivalent (MET) h/week, representing the level of oxygen required to maintain resting metabolism. MET-h/week was calculated by summing the products of the duration (hours per week) and the MET-intensity value for each reported physical activity, based on a validated questionnaire and standard MET reference values. Before the analysis, educational levels were categorized into three groups: primary school or below, junior high school, and senior high school or above. Annual household income was divided into four categories: low income (less than 30,000 yuan), middle income (30,000–75,000 yuan), high income (75,000–120,000 yuan), and very high income (more than 120,000 yuan). Smoking status and drinking status were each divided into two groups: smokers were defined as those who currently smoking or with a history of smoking, and drinkers were defined as those who had consumed beer, liquor, or other alcoholic beverages in the past year. Body mass index (BMI) was calculated by dividing the body weight (kg) by height squared (m^2^). Type 2 diabetes mellitus (T2DM) was defined by FBG level ≥ 7.0 mmol/L or HbA1c ≥ 6.5% or prior treatment for diabetes. Hypertension was defined by blood pressure ≥ 140/90 mmHg or use of antihypertensive medication. UCr levels were utilized to correct for urine dilution in cadmium exposure assessments. Missing covariates were handled using the multiple imputation method.

### 2.6. Statistical Analysis

Continuous variables were described using the mean and standard deviation (SD) or median and interquartile range (IQR), while categorical variables were described in terms of frequency and percentage (%). The *t*-test, Wilcoxon rank-sum test, and the Chi-square test were used to compare characteristics between male and female participants. Spearman’s correlation analysis was used to calculate the correlation coefficients between UCd exposure and serum minerals (Figure S1). Due to right-skewed distributions, natural logarithmic transformation (Ln) was applied to serum Fe, Se, P, UCd, and covariates, including physical activity, total energy intake, total fat intake, BMI, and UCr before analysis. Serum minerals, UCd, and MMSE score underwent z-score standardization to enable comparative effect size interpretation.

#### 2.6.1. Single-Exposure Association Analysis

Multiple linear regression assessed associations of urinary cadmium (UCd) and serum minerals (analyzed both as continuous variables and categorized into quartiles) with MMSE score. Linear trends across quartiles of serum minerals were examined by assigning the median value to each quartile and treating this as a continuous variable in the regression model, with the resulting *p*-value reported as the P for trend.

#### 2.6.2. Effect Modification Analysis

Joint exposure groups were defined by median splits of UCd and each mineral (high/low). Linear models compared MMSE differences relative to the high-UCd/low-mineral reference group. Multiplicative interaction terms (UCd × mineral) were tested for effect modification. To account for multiple comparisons across mineral groups and reduce the risk of type I error, False Discovery Rate (FDR) correction was applied to all corresponding *p*-values derived from joint grouping and interaction analyses within the total population.

#### 2.6.3. Exposure-Response Analysis

Restricted cubic splines (RCS) with knots at the 10th, 50th, and 90th percentiles characterized nonlinear dose–response relationships between individual exposures (UCd/minerals) and MMSE score.

#### 2.6.4. Stratified Analysis

Heterogeneity of associations was examined across demographic strata defined by age (<65/≥65 years), gender (male/female), and educational attainment (junior high school or below/senior high school or above), with interaction *p* values derived from likelihood ratio tests. All *p*-values from subgroup-specific joint exposure analyses and interaction analyses were further adjusted for multiple testing using the FDR method within each stratification group.

#### 2.6.5. Mixed-Exposure Association Analysis

The Quantile g-computation (QGC) model estimates the overall mixture effect and component weights by transforming mixed exposures into quantiles and constructing a weighted exposure index, employing the parametric g-formula. Bootstrap resampling (1000 repetitions) was used to derive 95% confidence intervals, with joint effects inferred via linear or generalized linear regression models [32]. The QGC models were applied to (i) estimate the joint effects of cadmium-mineral mixtures and of the mixture of five minerals alone on MMSE score, and (ii) compare these joint effects against cadmium-only exposure.

#### 2.6.6. Sensitivity Analysis

To ensure the robustness of our findings, we conducted four sensitivity analyses: (1) repeating linear regression analyses for single exposures (UCd, minerals) and MMSE after excluding individuals with UCd or serum mineral concentrations above the 99th percentile; (2) analyzing the dataset without multiple imputation for missing data; (3) reanalyzing the main outcomes after excluding participants with T2DM or hypertension; and (4) examining the association between urinary cadmium exposure and cognitive function using serum cadmium concentration as an alternative biomarker.

The covariates adjusted in the models as shown in the directed acyclic graph, included age (continuous), gender (binary), residence (binary), education level (categorical), annual household income (categorical), smoking (binary), drinking (binary), physical activity (continuous), total daily energy intake (continuous), total daily fat intake (continuous), BMI (continuous), UCr (continuous), T2DM (binary), and hypertension (binary; Figure S2). All statistical models, including linear regression, quantile regression, interaction analyses, and QGC, were adjusted for the covariates listed above, except that gender was not adjusted for in gender-stratified subgroups, age was not adjusted for in age-stratified subgroups, and educational level was not adjusted for in education-stratified subgroups, respectively. Statistical analyses were performed using R (version 4.2.2). The QGC was implemented using the “qgcomp” package. A two-sided *p* value < 0.05 was considered statistically significant.

## 3. Results

### 3.1. Participant Characteristics

The basic characteristics of the 6795 study participants are presented in Table 1. The mean age of the participants was 57.96 ± 10.46 years, with a higher proportion of females than males. Compared to females, males had a higher prevalence of education levels at junior high school, high school, or above, were more likely to be smokers and alcohol drinkers, had a higher prevalence of hypertension, and reported higher total energy and fat intake (*p* < 0.05). Males also had higher urinary creatinine levels. The mean MMSE score was 0.91 points higher in males than in females. Serum Fe levels were significantly higher in males, while serum P levels were lower. The mean UCd exposure level was 0.06 µg/L higher in males than in females. Spearman correlation coefficients between UCd and the five serum minerals varied between −0.14 and 0.40 (Figure S1), with the strongest negative correlation observed between UCd and P (ρ = –0.14, *p* < 0.001). Among the minerals themselves, Ca showed the strongest pairwise correlation with Mg (ρ = 0.40, *p* < 0.001), followed by its associations with Fe and P (ρ = –0.12, *p* < 0.001), while correlations among other mineral pairs were generally weak.

### 3.2. Associations of UCd Exposure and Minerals with MMSE

After adjusting for potential confounders, linear regression models revealed an inverse association between elevated UCd concentration and MMSE score (β = −0.035; 95% CI: −0.057, −0.013). Conversely, Ca, Mg, P, and Se showed positive associations with MMSE. Regression analysis based on quartiles demonstrated that compared to the first quartile (Q1), the fourth quartile (Q4) of UCd was inversely associated with MMSE score. Compared to Q1, Q2 of Fe was positively associated with MMSE score (Table 2).

**Figure 2:**
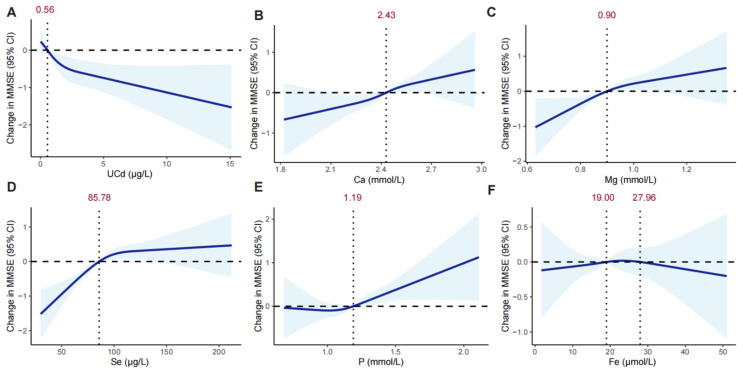
Nonlinear associations of urinary cadmium and serum minerals with MMSE Scores using restricted cubic splines. (**A**) UCd; (**B**) Ca; (**C**) Mg; (**D**) Se; (**E**) P; (**F**) Fe. Abbreviations: MMSE, mini-mental state examination; UCd, urinary cadmium; Ca, calcium; Fe, ferrum; Mg, magnesium; P, phosphorus; Se, selenium. Models were adjusted for age (continuous), sex (binary), residence (categorical), education level (categorical), household income (categorical), smoking (binary), alcohol drinking (binary), physical activity (continuous), total energy intake (continuous), total fat intake (continuous), BMI (continuous), T2DM (binary), hypertension (binary).

RCS models indicated a linear decreasing trend in the association between UCd and MMSE score. In contrast, associations between Ca and Mg with MMSE score exhibited linearly increasing trends. For P, no significant change in MMSE score was observed at lower concentrations, whereas a linearly increasing trend emerged beyond 1.19 mmol/L. Conversely, Se exhibited a linearly increasing association with MMSE score below 85.78 μg/L, with no significant association detected at higher concentrations. The association between Fe and MMSE score exhibited a complex pattern, characterized by an initial decrease, followed by an increase, and then another decrease (Figure 2).

### 3.3. Modifying Effects of Minerals on Cadmium-Related Cognitive Decline

Joint grouping analysis compared MMSE scores across groups defined by UCd and each mineral relative to the high UCd/low mineral reference group. For Ca and Fe, both the low UCd/high mineral and low UCd/low mineral groups were associated with significantly higher MMSE scores. A similar pattern was observed for Mg, P, and Se, where the low UCd groups with either high or low mineral levels also showed significantly higher scores. Notably, even under high cadmium exposure, groups with high levels of Mg, P, or Se remained associated with increased MMSE scores, suggesting a specific protective antagonism against cadmium neurotoxicity for these minerals. However, including multiplicative interaction terms in the models revealed no statistically significant interactions between UCd and any of the five minerals (Table 3).

### 3.4. Stratified Analysis

In subgroup analyses stratified by gender, a significant inverse association between UCd exposure and MMSE score was observed in females (β = −0.049; 95% CI: −0.080, −0.017), but not in males. Positive associations between Ca, Mg, Fe, P, and Se with MMSE score were found in females. In males, only Mg and Se showed positive linear associations with MMSE score (Table S2). Joint grouping analysis within gender strata indicated that, compared to the high UCd/low minerals group, the high UCd/high Ca group (β = 0.13; 95% CI: 0.04, 0.22) and the high UCd/high P group (β = 0.19; 95% CI: 0.10, 0.28) were positively associated with MMSE score only in females. The high UCd/high Se group was associated with increased MMSE score in both genders. No significant mineral-cadmium interactions were observed between male and female subgroups (Table S3).

In age-stratified analyses (40–65 years vs. ≥65 years), Ca, Mg, and Se were positively associated with MMSE score in the middle-aged group (40–65 years). In the elderly group (≥65 years), P showed an additional positive association. Compared to the high UCd/low minerals group, the high UCd/high Ca group (β = 0.21; 95% CI: 0.02, 0.40) and the high UCd/high Mg group (β = 0.21; 95% CI: 0.03, 0.40) were positively associated with MMSE score only in the elderly. The high UCd/high Se group was associated with increased MMSE score in both age groups. A significant interaction between P and UCd was observed in the middle-aged subgroup (Tables S4 and S5).

In analyses stratified by education level (junior high school or below vs. senior high school or above), a significant inverse association between UCd exposure and MMSE score was found in the lower education group (β = −0.044; 95% CI: −0.074, −0.015), but not in the higher education group. In the higher education group, Fe, Mg, and Se were positively associated with MMSE score. Ca, Mg, P, and Se showed positive associations among people with junior high school or below education level. Compared to the high UCd/low minerals group, the high UCd/high P group (β = 0.10; 95% CI: 0.02, 0.18) was positively associated with MMSE score only in the lower education group. The high UCd/high Se group was associated with increased MMSE score in both education groups. No significant mineral-cadmium interactions were found between the senior high school or above subgroup and the junior high school or below subgroup (Tables S6 and S7).

### 3.5. Joint Associations of UCd and Minerals with MMSE

**Figure 3:**
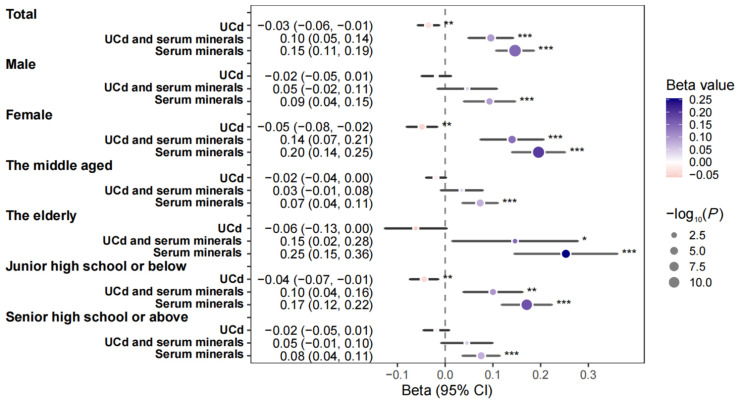
Associations of urinary cadmium and serum minerals with MMSE scores using linear regression and Quantile g-computation. ***: *p* < 0.001, **: *p* < 0.01, *: *p* < 0.05. Abbreviations: MMSE, mini-mental state examination; UCd, urinary cadmium. Models were adjusted for age (continuous), sex (binary), residence (categorical), education level (categorical), household income (categorical), smoking (binary), alcohol drinking (binary), physical activity (continuous), total energy intake (continuous), total fat intake (continuous), BMI (continuous), T2DM (binary), hypertension (binary), UCr (continuous).

Quantile g-computation (QGC) models revealed that the joint effect of UCd and the five minerals on MMSE score in the total population was positive (β = 0.10; 95% CI: 0.05, 0.14), but weaker than the joint effect of the five minerals alone (β = 0.15; 95% CI: 0.11, 0.19) (Figure 3). This attenuation suggests that cadmium may partially counteract the neuroprotective benefits of the mineral mixture, consistent with its known neurotoxicity and potential to disrupt mineral-dependent physiological processes. The joint association of UCd and the minerals with MMSE score was non-significant in the male, middle-aged (40–65 years), and higher education (high school or above) subgroups. Significant positive associations were observed in the female, elderly (≥65 years), and lower education (junior high school or below) subgroups. The joint effect of the five minerals on MMSE score was stronger in females (β = 0.20; 95% CI: 0.14, 0.25) than in males (β = 0.09; 95% CI: 0.04, 0.15). Similarly, the joint mineral effect was stronger in the elderly (β = 0.25; 95% CI: 0.15, 0.36) compared to the middle-aged (β = 0.07; 95% CI: 0.04, 0.11), and stronger in the lower education group (β = 0.17; 95% CI: 0.12, 0.22) than in the higher education group (β = 0.08; 95% CI: 0.04, 0.11).

### 3.6. Sensitivity Analysis

Sensitivity analyses confirmed the robustness of our primary linear regression models. After excluding individuals with UCd or serum mineral concentrations exceeding the 99th percentile, effect estimates remained consistent with main findings (Table S8). Re-analysis of the unimputed dataset yielded comparable results to those obtained with the imputed data (Table S9). Exclusion of participants with hypertension or diabetes did not alter the primary associations (Table S11). In supplementary analysis using serum cadmium (SCd) as the exposure metric, SCd demonstrated a significant inverse association with MMSE score (β = −0.024, 95% CI: −0.045, −0.003; Table S12), reinforcing the neurotoxic role of cadmium exposure.

## 4. Discussion

This nationwide study leverages prospective cohort data with delayed outcome assessment to systematically evaluate the modifying role of essential serum minerals in the association between baseline urinary cadmium exposure (2015) and cognitive decline measured three years later (2018). Our core findings demonstrate that elevated urinary cadmium (UCd) exposure is significantly associated with reduced MMSE score in middle-aged and elderly Chinese adults, corroborating prior evidence of cadmium’s neurotoxicity. More importantly, we identified five serum minerals—Ca, Fe, Mg, Se, and P—as beneficial factors for cognitive function. Notably, Mg, Se, and P demonstrated the strongest antagonistic effects against cadmium-related cognitive impairment.

The choice of biomarkers in this study was guided by their biological relevance and methodological stability in reflecting long-term exposure and nutritional status. Urinary cadmium (UCd) was selected as the primary exposure biomarker as it is widely regarded as a reliable indicator of long-term cadmium accumulation and body burden, particularly in populations with chronic low-level exposure. In contrast, blood cadmium reflects more recent exposure due to its shorter half-life [33]. Regarding the essential minerals, we measured their concentrations in serum rather than urine. This approach is grounded in the physiological principle that minerals such as calcium, magnesium, selenium, iron, and phosphorus are under tight homeostatic control in the blood, and their circulating levels are more likely to represent biologically active pools available for neurological processes. Urinary excretion of these minerals, on the other hand, is highly variable and influenced by recent dietary intake, renal function, and diurnal rhythms, making serum a more robust and clinically meaningful matrix for assessing nutritional status and its association with cognitive outcomes [18,19,34].

Similarly to a study conducted in older American adults, this study showed an inverse association between Cd and cognitive function in older adults, further corroborating the generalizability of Cd neurotoxicity across populations [35]. Furthermore, a growing body of evidence specifically associates cadmium exposure with neurodegenerative diseases such as Alzheimer’s disease. Several epidemiological studies have reported significantly higher cadmium levels in the blood and urine of Alzheimer’s patients compared to cognitively healthy individuals, suggesting cadmium may contribute to disease progression [7,8]. Consistent with this pattern, a study conducted in Bangladesh revealed a negative association between Cd and visual recognition and memory [36]. Another study in older US adults showed that blood Cd was negatively associated with all three cognitive tests, but urinary Cd was negatively associated with only one of the cognitive tests [37]. In addition, ecological studies have indicated a correlation between regional heavy metal pollution, with particular emphasis on cadmium, and higher incidence rates of Alzheimer’s disease, further underscoring the role of environmental neurotoxicants in cognitive aging [38]. Mechanistically, such population-level cognitive impairments may arise because Cd exposure may impair synaptic transmission, increase neuroinflammation, and disrupt mitochondrial integrity both structurally and functionally [39,40]. Cd has also been shown to promote Alzheimer’s-related pathological processes, including Aβ plaque deposition and microglial activation in the brain, providing a plausible biological basis for the observed epidemiological associations [9]. Collectively, these neurotoxic mechanisms ultimately manifest as measurable cognitive decline.

Previous epidemiological studies have primarily examined individual minerals in relation to cognitive outcomes, leaving a critical gap in understanding how combinations of essential minerals interact with neurotoxicants like Cd. Our QGC model addresses this limitation by providing the first population-level quantification of the protective multi-mineral antagonism against Cd neurotoxicity. The significantly attenuated effect of the Cd-mineral mixture compared to the mineral-only mixture demonstrates that mineral co-exposure substantially mitigates Cd’s neurotoxic potential. This finding aligns with previous investigations showing that Se and Mg counteract Cd-related oxidative stress and neuroinflammation, but extends beyond single-mineral paradigms to reveal clinically relevant synergistic protection [41,42].

The observed antagonism in our QGC results likely operates through complementary physiological pathways. As plenty of previous experimental studies and reviews have shown, Mg modulates NMDA receptor function and synaptic plasticity while activating BDNF signaling pathways critical for neuronal survival [42,43]. Se upregulates glutathione peroxidase activity to scavenge Cd-generated reactive oxygen species and promotes metallothionein synthesis for Cd sequestration [41,44]. Ca competitively inhibits Cd uptake via the divalent metal transporter 1 (DMT1) in enterocytes and stabilizes neuronal calcium signaling [45,46]. P supports adenosine triphosphate-dependent neuroenergetics, while iron enables oxygen transport and myelin synthesis [47,48]. Moreover, Fe and Cd share similar absorption mechanisms, and elevated levels of Fe in the body may facilitate more efficient elimination of Cd [49]. The identified mechanisms corroborate clinical evidence across diverse populations. Hypomagnesemia and hypocalcemia associate with accelerated cognitive decline in hospitalized elderly, while Mg and P exert protective effects in hemodialysis patients, who are susceptible to Cd exposure [50,51]. This consistency across experimental, clinical, and epidemiological evidence strengthens the biological plausibility of our findings, though the synergistic multi-mineral protection quantified herein represents a novel contribution to the field.

Our restricted cubic spline analysis revealed mineral-specific neuroprotective dynamics distinct from linear dose–response assumptions. P exhibited a threshold effect (no cognitive protection below 1.19 mmol/L), whereas Se demonstrated a saturation effect (diminishing returns above 85.78 μg/L). Consistent with our findings, Se above nutritionally essential levels may also alter circulating Se levels and endogenous selenoprotein production, thereby negatively affecting various neurophysiological functions [34]. However, hyperphosphatemia is a risk factor for vascular calcification, and elevated phosphate levels beyond the physiological range directly affect endothelial function and vascular remodeling, with concomitant vascular and degenerative brain injury increasing the risk of dementia by more than 2-fold [19]. These nonlinear patterns refine clinical understanding by establishing optimal protective ranges. Such nuances explain inconsistencies in prior single-mineral studies that overlooked concentration-dependent efficacy.

The absence of significant multiplicative interactions despite the observed protective patterns in high-mineral subgroups suggests the presence of nonlinear or threshold-dependent mechanisms, rather than simple effect modification. Such complex dynamics are consistent with the mineral-specific neuroprotective patterns identified in our restricted cubic spline analyses. The protective effects specific to Mg, P, and Se, but not Ca or Fe, may be attributed to their distinct roles in mitigating cadmium toxicity. For instance, animal studies have confirmed the antagonistic interaction between cadmium and magnesium, likely attributable to excessive magnesium intake inhibiting cadmium transport through intestinal channels and thereby reducing its gastrointestinal absorption [52]. Selenium is known to form inert cadmium-selenide complexes, thereby reducing bioavailable cadmium levels [41]. Phosphorus, which is critical for cellular energy metabolism and structural integrity, may enhance neuronal resilience under cadmium-induced metabolic stress [47].

Stratified analyses revealed critical demographic heterogeneity, it reveals the key public health problem worthy of attention. The UCd-cognition association was markedly stronger in females and individuals with lower education, potentially reflecting hormonal influences, differential cadmium metabolism, or cognitive reserve limitations [53]. Notably, Mg and Se conferred consistent protection across all subgroups, suggesting their role as universal neuroprotective agents. Inconsistent with our study, a study of an elderly population over 60 years of age in the United States showed that blood Se concentration was positively associated with cognitive performance only in men, whereas no association was observed in women [54]. The observed gender-specific efficacy of Ca and Fe in females may stem from hormonal modulation of nutrient metabolism (e.g., estrogen’s role in enhancing calcium retention for bone health) and higher baseline nutrient demands in premenopausal women due to menstrual iron losses [55,56]. Furthermore, our study found Fe provided targeted benefits for the elderly (≥65 years), while Ca and P provided health gains for low-education groups. The benefit of Fe in the elderly likely reflects age-related impairments in nutrient absorption (reduced gastric acid production limiting non-heme iron uptake) and increased anemia susceptibility [57]. These findings highlight the need for precision nutrition strategies tailored to population vulnerabilities.

This study possesses several notable strengths. Firstly, it leveraged a large, nationally representative cohort encompassing participants from more than 200 research sites across 15 provinces in China, significantly enhancing the generalizability of the findings. Secondly, a key methodological innovation was the novel application of QGC modeling, which allowed us to quantify, for the first time, the magnitude of the protective antagonistic effect exerted by the joint exposure to essential minerals against Cd toxicity. Furthermore, our comprehensive assessment of different subpopulations revealed important variations in susceptibility and protective effects, identifying vulnerable subgroups (e.g., females, those with lower education) and broadly protective minerals (e.g., Mg, Se).

However, several limitations warrant consideration. Firstly, although cognitive function was assessed three years after exposure measurement, the absence of baseline cognitive data precludes definitive causal inference. We cannot exclude reverse causation, whereby subclinical cognitive decline at exposure baseline might influence mineral metabolism or cadmium accumulation. Secondly, reliance on the MMSE, while practical, may have led to underestimation of subtle cognitive changes, particularly mild cognitive impairment involving executive function, due to its known suboptimal sensitivity in this domain. Thirdly, urinary Cd reflects recent exposure and body burden but may not fully represent long-term cumulative exposure or target organ accumulation, and single-point serum mineral measurements might not capture long-term nutritional status. Fourthly, although the sample sizes in most stratified subgroups were sufficient and well-balanced, we acknowledge that some finely stratified analyses, particularly those involving high cadmium and high mineral concentrations, may have had limited statistical power to detect subtle interaction effects. Therefore, the null interactions observed in certain subgroups should be interpreted with caution and confirmed in future studies with larger sample sizes. Despite comprehensive adjustment for major confounders, residual confounding from unmeasured or imperfectly measured factors (e.g., genetic susceptibility, other environmental neurotoxicants) cannot be entirely ruled out. Finally, this study may be limited by selection bias due to the exclusion of participants under 40 years of age or with incomplete biomarker data. This may affect the generalizability of the findings to populations with differing dietary patterns, environmental exposures, or genetic backgrounds.

## 5. Conclusions

This study demonstrates that urinary cadmium (Cd) exposure is an independent risk factor for cognitive decline in middle-aged and older adults. Critically, we observed that essential minerals—calcium (Ca), iron (Fe), magnesium (Mg), selenium (Se), and phosphorus (P)—confer significant protection against Cd-associated cognitive impairment, with the combined protective effect of the mineral mixture being particularly significant. Notably, the strength of this beneficial effect exhibited population heterogeneity. Our findings provide robust translational evidence: Maintaining optimal serum levels of Mg, Se, P, Ca, and Fe, especially in cadmium-exposed females, elderly individuals, and populations with lower educational attainment, could significantly mitigate cognitive decline. These results offer a scientific basis for developing targeted nutritional strategies and environmental health policies in high Cd-exposure populations.

## Figures and Tables

**Table 1:** Characteristics of the included participants in CHNS 2015.

	Total (n = 6795)	Male (n = 3090)	Female (n = 3705)	*p* Value
Age, year	57.96 (10.46)	58.26 (10.50)	57.71 (10.43)	0.033
Rural, n (%)	2512 (37.0)	1117 (36.1)	1395 (37.7)	0.210
**Education, n (%)**				<0.001
Primary school or below	4761 (70.1)	2017 (65.3)	2744 (74.1)	
Junior high school	1431 (21.1)	737 (23.9)	694 (18.7)	
Senior high school or above	603 (8.9)	336 (10.9)	267 (7.2)	
**Annual household income, yuan (%)**				0.310
Low	2803 (41.3)	1237 (40.0)	1566 (42.3)	
Medium	2378 (35.0)	1099 (35.6)	1279 (34.5)	
High	976 (14.4)	457 (14.8)	519 (14.0)	
Very high	638 (9.4)	297 (9.6)	341 (9.2)	
T2DM, n (%)	829 (12.2)	395 (12.8)	434 (11.7)	0.192
Hypertension, n (%)	2943 (43.3)	1412 (45.7)	1531 (41.3)	<0.001
Smoker, n (%)	1815 (26.7)	1737 (56.2)	78 (2.1)	<0.001
Alcohol user, n (%)	1893 (27.9)	1672 (54.1)	221 (6.0)	<0.001
Physical activity, MET h/week	103.75 [47.43, 209.60]	112.30 [43.52, 226.50]	98.00 [48.71, 196.93]	0.129
Total energy intake, kcal	1912.59 [1514.39, 2422.72]	2092.07 [1668.00, 2661.64]	1761.91 [1407.69, 2212.75]	<0.001
Total fat intake, g	71.91 [51.06, 100.99]	79.21 [55.84, 109.92]	65.90 [47.65, 92.73]	<0.001
BMI, kg/m^2^	24.27 [22.07, 26.61]	24.20 [21.94, 26.58]	24.32 [22.17, 26.70]	0.058
Urinary creatinine, μmol/L/24 h	5676.00 [3249.00, 9123.50]	6643.50 [3906.00, 10,439.00]	4973.00 [2808.00, 8003.00]	<0.001
MMSE score	27.12 (4.78)	27.62 (4.26)	26.71 (5.14)	<0.001
Ca, mmol/L	2.42 (0.14)	2.42 (0.14)	2.42 (0.14)	0.060
Mg, mmol/L	0.91 (0.09)	0.91 (0.09)	0.90 (0.08)	<0.001
Fe, μmol/L	19.00 [14.90, 23.80]	21.30 [16.80, 26.60]	17.40 [13.70, 21.40]	<0.001
P, mmol/L	1.19 [1.07, 1.30]	1.12 [1.01, 1.23]	1.24 [1.13, 1.35]	<0.001
Se, μg/L	85.78 [72.70, 98.62]	85.82 [73.09, 98.90]	85.76 [72.26, 98.32]	0.413
UCd, μg/L	0.56 [0.27, 1.22]	0.60 [0.28, 1.27]	0.54 [0.26, 1.18]	0.010

Abbreviations: CHNS, China Health and Nutrition Survey; T2DM, type 2 diabetes mellitus; MET, metabolic equivalent; BMI, body mass index; MMSE, mini-mental state examination; Ca, calcium; Fe, ferrum; Mg, magnesium; P, phosphorus; Se, selenium; UCd, urinary cadmium. Data were presented as the mean (SD), median [IQR], or frequency (percentage).

**Table 2:** Linear model results for the association of urinary cadmium and serum minerals with MMSE in general population.

Exposure	Beta (95% CI)	Q1	Q2	Q3	Q4	*P*_-trend_
UCd	−0.035 (−0.057, −0.013)	Ref.	0.00 (−0.06, 0.06)	−0.05 (−0.11, 0.01)	−0.09 (−0.15, −0.02)	0.003
Ca	0.042 (0.020, 0.064)	Ref.	0.06 (0.00, 0.12)	0.10 (0.04, 0.16)	0.10 (0.04, 0.16)	<0.001
Fe	0.014 (−0.008, 0.036)	Ref.	0.06 (0.00, 0.12)	0.05 (−0.01, 0.11)	0.05 (−0.02, 0.11)	0.199
Mg	0.053 (0.031, 0.074)	Ref.	0.11 (0.05, 0.17)	0.13 (0.07, 0.19)	0.15 (0.09, 0.21)	<0.001
P	0.041 (0.019, 0.064)	Ref.	0.01 (−0.05, 0.07)	0.06 (0.00, 0.12)	0.12 (0.05, 0.18)	<0.001
Se	0.080 (0.058, 0.102)	Ref.	0.14 (0.07, 0.20)	0.22 (0.15, 0.28)	0.20 (0.14, 0.26)	<0.001

Models were adjusted for age (continuous), sex (binary), residence (categorical), education level (categorical), household income (categorical), smoking (binary), alcohol drinking (binary), physical activity (continuous), total energy intake (continuous), total fat intake (continuous), BMI (continuous), T2DM (binary), hypertension (binary). Abbreviations: MMSE, mini-mental state examination; UCd, urinary cadmium; Ca, calcium; Fe, ferrum; Mg, magnesium; P, phosphorus; Se, selenium; BMI, body mass index; T2DM, type 2 diabetes mellitus. The quartile cut-offs for each exposure are based on untransformed concentrations and are provided in their original units for clinical context: UCd Q1: <0.27 μg/L, Q2: 0.27~0.56 μg/L, Q3: 0.56~1.22 μg/L, Q4: ≥1.22 μg/L; Ca Q1: <2.34 mmol/L, Q2: 2.34~2.43 mmol/L, Q3: 2.43~2.50 mmol/L, Q4: ≥2.50 mmol/L; Fe Q1: <14.90 μmol/L, Q2: 14.90~19.00 μmol/L, Q3: 19.00~23.80 μmol/L, Q4: ≥23.80 μmol/L; Mg Q1: <0.85 mmol/L, Q2: 0.85~0.90 mmol/L, Q3: 0.90~0.96 mmol/L, Q4: ≥0.96 mmol/L; P Q1: <1.07 μmol/L, Q2: 1.07~1.19 μmol/L, Q3: 1.19~1.30 μmol/L, Q4: ≥1.30 μmol/L; Se Q1: <72.70 μg/L, Q2: 72.70~85.78 μg/L, Q3: 85.78~98.62 μg/L, Q4: ≥98.62 μg/L.

**Table 3:** Results of the combined group regression model for urinary cadmium and serum minerals in relation to MMSE scores in the general population.

Minerals	Exposure	Beta (95% CI)				*P*_-interaction_
		High UCd and Low Minerals	Low UCd and Low Minerals	High UCd and High Minerals	Low UCd and High Minerals	
Ca	UCd	Reference	0.08 (0.02, 0.15)	0.06 (0.00, 0.12)	0.16 (0.09, 0.22)	0.749
Fe	UCd	Reference	0.10 (0.03, 0.16)	0.02 (−0.04, 0.08)	0.11 (0.05, 0.18)	0.853
Mg	UCd	Reference	0.08 (0.01, 0.14)	0.07 (0.01, 0.13)	0.18 (0.11, 0.24)	0.749
P	UCd	Reference	0.10 (0.04, 0.17)	0.09 (0.03, 0.16)	0.17 (0.10, 0.23)	0.749
Se	UCd	Reference	0.11 (0.05, 0.18)	0.15 (0.09, 0.21)	0.25 (0.18, 0.32)	0.749

Models were adjusted for age (continuous), sex (binary), residence (categorical), education level (categorical), household income (categorical), smoking (binary), alcohol drinking (binary), physical activity (continuous), total energy intake (continuous), total fat intake (continuous), BMI (continuous), T2DM (binary), hypertension (binary). Abbreviations: MMSE, mini-mental state examination; UCd, urinary cadmium; Ca, calcium; Fe, ferrum; Mg, magnesium; P, phosphorus; Se, selenium; BMI, body mass index; T2DM, type 2 diabetes mellitus. The *P*_-interaction_ values were adjusted for multiple testing using the FDR method.

## Data Availability

The datasets used and/or analyzed during the current study are available from the corresponding author upon reasonable request.

## Supplementary Materials

The following supporting information can be downloaded at: https://www.mdpi.com/article/10.3390/nu17182910/s1, Method S1: Determination of urinary cadmium and serum selenium by ICP-MS; Figure S1: Spearman correlations among the concentrations of urinary cadmium exposure and serum essential minerals; Figure S2: The directed acyclic graph of the association between urinary cadmium and MMSE; Table S1: The concentration distribution of urinary cadmium exposure and serum essential minerals; Table S2: Linear model results for the association of urinary cadmium and serum minerals with MMSE for gender subgroups; Table S3: Results of the combined group regression model for urinary cadmium and serum minerals for gender subgroups; Table S4: Linear model results for the association of urinary cadmium and serum minerals with MMSE for age subgroups; Table S5: Results of the combined group regression model for urinary cadmium and serum minerals for age subgroups; Table S6: Linear model results for the association of urinary cadmium and serum minerals with MMSE for subgroups of educational level; Table S7: Results of the combined group regression model for urinary cadmium and serum minerals for subgroups of educational level; Table S8: Association of urinary cadmium and serum minerals with MMSE in overall population excluding all individuals with concentrations of urinary cadmium or minerals above the 99th percentile; Table S9: Association of urinary cadmium and serum minerals with MMSE in overall population without multiple interpolation of covariates; Table S10: Characteristics of participants included in CHNS 2015 excluding those with T2DM or hypertension; Table S11: Association of urinary cadmium and serum minerals with MMSE in overall population excluding patients with hypertension and type 2 diabetes mellitus; Table S12: Association of serum cadmium and minerals with MMSE in overall population.

## Abbreviations

The BMI, body mass index; Ca, calcium; CHNS, China Health and Nutrition Survey; Fe, ferrum; FBG, fasting blood glucose; HbA1c, glycated hemoglobin A1c; ICP-MS, inductively coupled plasma-Mass Spectrometry; IQR, interquartile range; LLOQ, lower limit of quantitation; Ln, ln-transformed; MET, metabolic equivalent; Mg, magnesium; MMSE, mini-mental state examination; P, phosphorus; QGC, Quantile g-computation; RCS, Restricted cubic spline; SCd, serum cadmium; Se, selenium; SD, standard deviation; T2DM, type 2 diabetes mellitus; UCd, urinary cadmium.
