# Supplementary material for: Protective Role of Multiple Essential Minerals Against Cadmium-Related Cognitive Decline in Middle-Aged and Older Adults: A Prospective Study
Source: Nutrients. 2025 Sep 9;17(18):2910. doi: 10.3390/nu17182910 (PMC12473016; doi:10.3390/nu17182910)
Supplement: Supplementary file 1 [file nutrients-17-02910-s001.zip › nutrients-3809729-supplementary.pdf]

Supplementary Materials

Protective role of multiple essential minerals against cadmium-related cognitive decline in middle-aged and older adults: A prospective study

Jing Yang [a †], Zongyao Li [b,c †], Yongbin Zhao [d,e,f], Yanzhen Hu [b,c], Xinyang Guo [b,c], Xi Kang [g,h], Zhenyu Wu [i], Chang Su [g,h *], Tao Zhang [d,e,f *]

[a] Chinese Center for Disease Control and Prevention, Beijing 102206, China

[b] Department of Biostatistics, School of Public Health, Cheeloo College of Medicine, Shandong University, Jinan 250012, China

[c] Institute for Medical Dataology, Shandong University, Jinan 250002, China

[d] Department of Epidemiology and Statistics, School of Public Health, Tianjin Medical University, Tianjin 300070, China

[e] Tianjin Key Laboratory of Environment, Nutrition and Public Health, Tianjin 300070, China

[f] Key Laboratory of Prevention and Control of Major Diseases in the Population, Ministry of Education, Tianjin Medical University, Tianjin 300070, China

[g] National Institute for Nutrition and Health, Chinese Center for Disease Control and Prevention, Beijing 100050, China

[h] Key Laboratory of Public Nutrition and Health, National Health Commission of the People's Republic of China, Beijing 100050, China

[i] School of Public Health, Fudan University, Shanghai 200032, China

Table of Contents

Method S1: Determination of urinary cadmium and serum selenium by ICP-MS

Figure S1. Spearman correlations among the concentrations of urinary cadmium exposure and serum essential minerals.

Figure S2. The directed acyclic graph of the association between urinary cadmium and MMSE.

Table S1. The concentration distribution of urinary cadmium exposure and serum essential minerals.

Table S2. Linear model results for the association of urinary cadmium and serum minerals with MMSE for gender subgroups.

Table S3. Results of the combined group regression model for urinary cadmium and serum minerals for gender subgroups.

Table S4. Linear model results for the association of urinary cadmium and serum minerals with MMSE for age subgroups.

Table S5. Results of the combined group regression model for urinary cadmium and serum minerals for age subgroups.

Table S6. Linear model results for the association of urinary cadmium and serum minerals with MMSE for subgroups of educational level.

Table S7. Results of the combined group regression model for urinary cadmium and serum minerals for subgroups of educational level.

Table S8. Association of urinary cadmium and serum minerals with MMSE in overall population excluding all individuals with concentrations of urinary cadmium or minerals above the 99th percentile.

Table S9. Association of urinary cadmium and serum minerals with MMSE in overall population without multiple interpolation of covariates.

Table S10. Characteristics of participants included in CHNS 2015 excluding those with T2DM or hypertension.

Table S11. Association of urinary cadmium and serum minerals with MMSE in overall population excluding patients with hypertension and type 2 diabetes mellitus.

Table S12. Association of serum cadmium and minerals with MMSE in overall population.

Method S1: Determination of urinary cadmium and serum selenium by ICP-MS

Urinary cadmium (UCd) and serum selenium concentrations were determined using an inductively coupled plasma mass spectrometer (ICP-MS; Agilent 7700 series, Agilent Technologies, USA). Serum samples were prepared for selenium analysis using the standard addition method: Briefly, 100 μL of serum was diluted with 1900 μL of diluent (containing 0.1000% (v/v) nitric acid and 0.1000% (v/v) Triton X-100 in ultrapure water) prior to ICP-MS analysis. Urinary cadmium (UCd) analysis employed the same standard addition preparation method: 200 μL of urine was diluted with 1800 μL of the same diluent (0.1000% (v/v) nitric acid and 0.1000% (v/v) Triton X-100 in ultrapure water) for ICP-MS analysis. The nitric acid used was HNO_3, 65% (v/v) (CFEQ-4-110040-0501, CNW), and Triton X-100 was obtained from Sigma-Aldrich (X100-500mL). Method accuracy and precision were monitored using commercially available certified reference materials. Serum selenium analysis utilized ClinChek® Human Serum Controls Levels I & II (Recipe, Cat. Nos. 8880 & 8881), while urinary cadmium analysis utilized ClinChek® Human Urine Controls Levels I & II (Recipe, Cat. Nos. 8847 & 8848). All measured values for the reference materials fell within their respective certified ranges. Inter-assay and intra-assay coefficients of variation (CVs) were maintained below 5% for selenium and below 8% for cadmium across all batches, in accordance with standard laboratory protocols.

Supplemental Figures

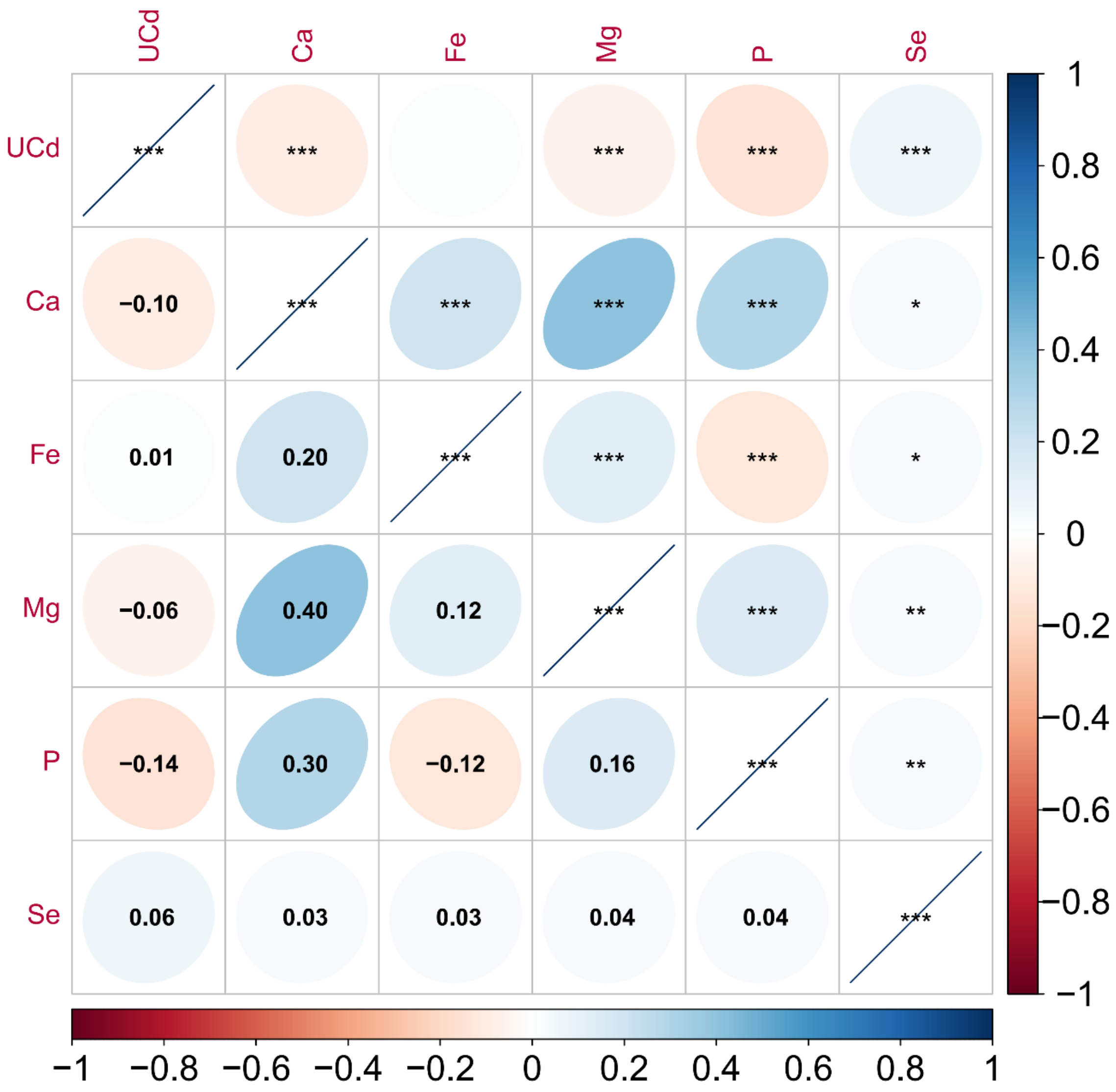

Figure S1. Spearman correlations among the concentrations of urinary cadmium exposure and serum essential minerals.

Abbreviations: UCd, urinary cadmium; Ca, calcium; Fe, ferrum; Mg, magnesium; P, phosphorus; Se, selenium.

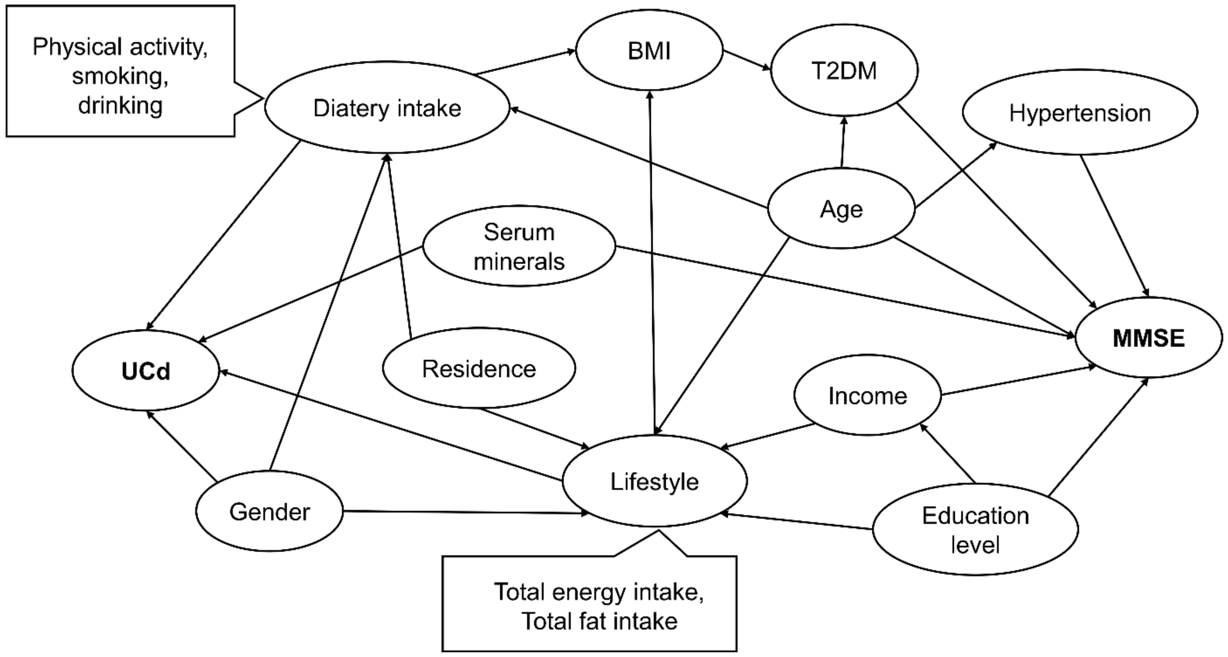

Figure S2. The directed acyclic graph of the association between urinary cadmium and MMSE.

Abbreviations: UCd, urinary cadmium; BMI, body mass index; T2DM, type 2 diabetes mellitus; MMSE, mini-mental state examination.

Supplemental Tables

Table S1. The concentration distribution of urinary cadmium exposure and serum essential minerals.

Family	Elements	LLOQ[a]	Detection rate (%)	Percentile				
				5th	25th	Median	75th	95th
Urine heavy metal	Cd (µg/L)	0.030	100.00	0.090	0.270	0.560	1.220	3.310
Serum minerals	Ca (mmol/L)	0.500	94.19	2.200	2.340	2.430	2.500	2.630
	Fe (µmol/L)	0.100	93.58	8.800	14.900	19.000	23.800	32.400
	Mg (mmol/L)	0.300	93.83	0.780	0.850	0.900	0.960	1.050
	P (mmol/L)	0.500	93.57	0.900	1.070	1.190	1.300	1.490
	Se (µg/L)	2.360	100.00	53.247	72.700	85.780	98.620	122.120

[a]Distribution of exposures among the 6795 participants in the analysis.

Abbreviations: LLOQ, lower limit of quantitation; Cd, cadmium; Ca, calcium; Fe, ferrum; Mg, magnesium; P, phosphorus; Se, selenium.

Table S2. Linear model results for the association of urinary cadmium and serum minerals with MMSE for gender subgroups.

Subgroups	Exposure	Beta (95%CI)	Q1	Q2	Q3	Q4	$P_{\text{-trend}}$
Male	UCd	-0.019 (-0.049, 0.012)	Ref.	-0.05 (-0.13, 0.03)	-0.06 (-0.14, 0.02)	-0.06 (-0.15, 0.03)	0.172
	Ca	0.014 (-0.016, 0.043)	Ref.	0.04 (-0.04, 0.12)	0.05 (-0.04, 0.13)	0.03 (-0.06, 0.11)	0.520
	Fe	0.016 (-0.016, 0.048)	Ref.	0.03 (-0.06, 0.13)	0.02 (-0.07, 0.11)	0.02 (-0.07, 0.11)	0.852
	Mg	0.040 (0.012, 0.069)	Ref.	0.13 (0.05, 0.22)	0.14 (0.06, 0.22)	0.13 (0.04, 0.21)	0.005
	P	0.015 (-0.014, 0.044)	Ref.	-0.04 (-0.11, 0.04)	0.00 (-0.09, 0.08)	0.06 (-0.03, 0.15)	0.276
	Se	0.075 (0.045, 0.105)	Ref.	0.09 (0.01, 0.18)	0.18 (0.10, 0.27)	0.17 (0.08, 0.25)	<0.001
Female	UCd	-0.049 (-0.080, -0.017)	Ref.	0.05 (-0.04, 0.13)	-0.04 (-0.13, 0.05)	-0.11 (-0.20, -0.02)	0.006
	Ca	0.075 (0.043, 0.106)	Ref.	0.08 (0.00, 0.17)	0.16 (0.07, 0.25)	0.19 (0.10, 0.27)	<0.001
	Fe	0.021 (-0.010, 0.052)	Ref.	0.08 (0.01, 0.16)	0.08 (0.00, 0.16)	0.07 (-0.02, 0.17)	0.075
	Mg	0.076 (0.044, 0.108)	Ref.	0.11 (0.03, 0.20)	0.15 (0.06, 0.23)	0.20 (0.11, 0.29)	<0.001
	P	0.093 (0.057, 0.128)	Ref.	0.12 (0.02, 0.23)	0.19 (0.09, 0.29)	0.25 (0.15, 0.35)	<0.001
	Se	0.086 (0.054, 0.118)	Ref.	0.17 (0.08, 0.26)	0.24 (0.15, 0.33)	0.24 (0.15, 0.33)	<0.001

Models were adjusted for age (continuous), sex (binary), residence (categorical), education level (categorical), household income (categorical), smoking (binary), alcohol drinking (binary), physical activity (continuous), total energy intake (continuous), total fat intake (continuous), BMI (continuous), T2DM (binary), hypertension (binary).

Abbreviations: MMSE, mini-mental state examination; UCd, urinary cadmium; Ca, calcium; Fe, ferrum; Mg, magnesium; P, phosphorus; Se, selenium; BMI, body mass index; T2DM, type 2 diabetes mellitus.

Table S3. Results of the combined group regression model for urinary cadmium and serum minerals for gender subgroups.

Subgroups	Minerals	Exposure	Beta (95%CI)				$P_{\text{-interaction}}$
			High UCd and low minerals	Low UCd and low minerals	High UCd and high minerals	Low UCd and high minerals	
Male	Ca	UCd	Reference	0.05 (-0.04, 0.14)	0.00 (-0.08, 0.08)	0.09 (0.00, 0.18)	0.955
	Fe	UCd	Reference	0.06 (-0.05, 0.16)	-0.01 (-0.09, 0.07)	0.07 (-0.03, 0.16)	0.955
	Mg	UCd	Reference	0.07 (-0.02, 0.17)	0.07 (-0.01, 0.15)	0.13 (0.04, 0.22)	0.955
	P	UCd	Reference	0.06 (-0.02, 0.14)	0.03 (-0.06, 0.11)	0.11 (0.02, 0.20)	0.955
	Se	UCd	Reference	0.10 (0.01, 0.19)	0.15 (0.07, 0.23)	0.21 (0.11, 0.30)	0.643
Female	Ca	UCd	Reference	0.11 (0.02, 0.20)	0.13 (0.04, 0.22)	0.23 (0.14, 0.32)	0.641
	Fe	UCd	Reference	0.11 (0.03, 0.20)	0.04 (-0.05, 0.13)	0.16 (0.06, 0.25)	0.641
	Mg	UCd	Reference	0.08 (-0.01, 0.17)	0.08 (-0.01, 0.17)	0.23 (0.13, 0.32)	0.641
	P	UCd	Reference	0.16 (0.06, 0.27)	0.19 (0.10, 0.28)	0.25 (0.16, 0.34)	0.127
	Se	UCd	Reference	0.12 (0.02, 0.21)	0.15 (0.06, 0.24)	0.29 (0.19, 0.38)	0.077

Models were adjusted for age (continuous), sex (binary), residence (categorical), education level (categorical), household income (categorical), smoking (binary), alcohol drinking (binary), physical activity (continuous), total energy intake (continuous), total fat intake (continuous), BMI (continuous), T2DM (binary), hypertension (binary), UCr (continuous). The $P_{\text{-interaction}}$ values were adjusted for multiple testing using the FDR method.

Abbreviations: MMSE, mini-mental state examination; UCd, urinary cadmium; Ca, calcium; Fe, ferrum; Mg, magnesium; P, phosphorus; Se, selenium; BMI, body mass index; T2DM, type 2 diabetes mellitus, UCr, urinary creatinine.

Table S4. Linear model results for the association of urinary cadmium and serum minerals with MMSE for age subgroups.

Subgroups	Exposure	Beta (95%CI)	Q1	Q2	Q3	Q4	$P_{\text{-trend}}$
Middle-aged adults (40-65 years)	UCd	-0.019 (-0.040, 0.002)	Ref.	0.01 (-0.05, 0.06)	0.00 (-0.06, 0.06)	-0.04 (-0.10, 0.01)	0.143
	Ca	0.034 (0.013, 0.055)	Ref.	0.05 (-0.01, 0.10)	0.06 (0.00, 0.12)	0.08 (0.03, 0.14)	0.005
	Fe	-0.010 (-0.030, 0.010)	Ref.	-0.01 (-0.07, 0.05)	-0.02 (-0.08, 0.04)	0.02 (-0.04, 0.08)	0.619
	Mg	0.031 (0.010, 0.051)	Ref.	0.05 (0.00, 0.11)	0.05 (0.00, 0.11)	0.07 (0.01, 0.13)	0.035
	P	0.016 (-0.005, 0.038)	Ref.	0.02 (-0.03, 0.08)	0.05 (-0.01, 0.10)	0.04 (-0.02, 0.10)	0.166
	Se	0.041 (0.019, 0.062)	Ref.	0.07 (0.01, 0.13)	0.14 (0.08, 0.20)	0.13 (0.07, 0.19)	<0.001
Older adults (≥65 years)	UCd	-0.061 (-0.126, 0.003)	Ref.	0.02 (-0.14, 0.19)	-0.13 (-0.30, 0.04)	-0.15 (-0.33, 0.03)	0.040
	Ca	0.080 (0.023, 0.137)	Ref.	0.09 (-0.07, 0.25)	0.21 (0.04, 0.38)	0.23 (0.05, 0.41)	0.004
	Fe	0.037 (-0.033, 0.106)	Ref.	0.15 (-0.01, 0.32)	0.09 (-0.08, 0.25)	0.04 (-0.15, 0.22)	0.803
	Mg	0.111 (0.049, 0.174)	Ref.	0.24 (0.07, 0.41)	0.29 (0.12, 0.45)	0.33 (0.15, 0.50)	<0.001
	P	0.090 (0.025, 0.154)	Ref.	-0.04 (-0.20, 0.13)	0.08 (-0.10, 0.26)	0.26 (0.07, 0.44)	0.004
	Se	0.142 (0.086, 0.199)	Ref.	0.27 (0.10, 0.44)	0.34 (0.17, 0.51)	0.34 (0.17, 0.51)	<0.001

Models were adjusted for age (continuous), sex (binary), residence (categorical), education level (categorical), household income (categorical), smoking (binary), alcohol drinking (binary), physical activity (continuous), total energy intake (continuous), total fat intake (continuous), BMI (continuous), T2DM (binary), hypertension (binary).

Abbreviations: MMSE, mini-mental state examination; UCd, urinary cadmium; Ca, calcium; Fe, ferrum; Mg, magnesium; P, phosphorus; Se, selenium; BMI, body mass index; T2DM, type 2 diabetes mellitus.

Table S5. Results of the combined group regression model for urinary cadmium and serum minerals for age subgroups.

Subgroups	Minerals	Exposure	Beta (95%CI)				*P*-interaction
			High UCd and low minerals	Low UCd and low minerals	High UCd and high minerals	Low UCd and high minerals	
Middle-aged adults (40-65 years)	Ca	UCd	Reference	0.03 (-0.03, 0.09)	0.03 (-0.03, 0.08)	0.10 (0.03, 0.16)	0.603
	Fe	UCd	Reference	0.05 (-0.01, 0.12)	0.01 (-0.05, 0.07)	0.05 (-0.01, 0.12)	0.774
	Mg	UCd	Reference	0.05 (-0.02, 0.11)	0.03 (-0.03, 0.08)	0.08 (0.02, 0.14)	0.275
	P	UCd	Reference	0.06 (0.00, 0.12)	0.04 (-0.02, 0.10)	0.08 (0.01, 0.14)	0.048
	Se	UCd	Reference	0.07 (0.01, 0.13)	0.11 (0.05, 0.17)	0.16 (0.10, 0.23)	0.872
Older adults (≥65 years)	Ca	UCd	Reference	0.26 (0.09, 0.43)	0.21 (0.02, 0.40)	0.40 (0.22, 0.58)	0.817
	Fe	UCd	Reference	0.23 (0.05, 0.41)	-0.03 (-0.22, 0.15)	0.23 (0.04, 0.42)	0.817
	Mg	UCd	Reference	0.25 (0.07, 0.43)	0.21 (0.03, 0.40)	0.43 (0.25, 0.61)	0.817
	P	UCd	Reference	0.22 (0.04, 0.40)	0.17 (-0.02, 0.37)	0.40 (0.22, 0.58)	0.817
	Se	UCd	Reference	0.27 (0.09, 0.46)	0.23 (0.05, 0.42)	0.47 (0.28, 0.66)	0.817

Models were adjusted for age (continuous), sex (binary), residence (categorical), education level (categorical), household income (categorical), smoking (binary), alcohol drinking (binary), physical activity (continuous), total energy intake (continuous), total fat intake (continuous), BMI (continuous), T2DM (binary), hypertension (binary), UCr (continuous). The P-interaction values were adjusted for multiple testing using the FDR method.

Abbreviations: MMSE, mini-mental state examination; UCd, urinary cadmium; Ca, calcium; Fe, ferrum; Mg, magnesium; P, phosphorus; Se, selenium; BMI, body mass index; T2DM, type 2 diabetes mellitus, UCr, urinary creatinine.

Table S6. Linear model results for the association of urinary cadmium and serum minerals with MMSE for subgroups of educational level.

Subgroups	Exposure	Beta (95%CI)	Q1	Q2	Q3	Q4	*P*-trend
Junior high school or below	UCd	-0.044 (-0.074, -0.015)	Ref.	0.00 (-0.08, 0.09)	-0.08 (-0.16, 0.00)	-0.10 (-0.18, -0.02)	0.003
	Ca	0.048 (0.020, 0.075)	Ref.	0.08 (0.00, 0.16)	0.14 (0.05, 0.22)	0.12 (0.04, 0.20)	0.001
	Fe	0.006 (-0.023, 0.036)	Ref.	0.06 (-0.02, 0.14)	0.05 (-0.03, 0.13)	0.02 (-0.07, 0.10)	0.726
	Mg	0.054 (0.026, 0.082)	Ref.	0.16 (0.08, 0.24)	0.15 (0.08, 0.23)	0.15 (0.07, 0.24)	<0.001
	P	0.061 (0.031, 0.091)	Ref.	0.01 (-0.07, 0.09)	0.10 (0.02, 0.18)	0.17 (0.08, 0.25)	<0.001
	Se	0.092 (0.064, 0.120)	Ref.	0.17 (0.09, 0.25)	0.26 (0.18, 0.33)	0.24 (0.16, 0.32)	<0.001
Senior high school or above	UCd	-0.019 (-0.045, 0.008)	Ref.	-0.01 (-0.07, 0.06)	0.00 (-0.07, 0.07)	-0.06 (-0.13, 0.02)	0.220
	Ca	0.021 (-0.007, 0.050)	Ref.	0.01 (-0.06, 0.08)	0.01 (-0.07, 0.08)	0.05 (-0.03, 0.12)	0.257
	Fe	0.027 (0.000, 0.054)	Ref.	0.06 (-0.02, 0.13)	0.05 (-0.03, 0.12)	0.10 (0.02, 0.17)	0.021
	Mg	0.044 (0.018, 0.070)	Ref.	0.00 (-0.08, 0.07)	0.07 (-0.01, 0.14)	0.11 (0.04, 0.19)	<0.001
	P	0.012 (-0.016, 0.039)	Ref.	0.01 (-0.06, 0.08)	-0.02 (-0.10, 0.06)	0.04 (-0.04, 0.12)	0.435
	Se	0.033 (0.003, 0.062)	Ref.	0.02 (-0.06, 0.10)	0.08 (0.00, 0.16)	0.08 (0.00, 0.16)	0.018

Models were adjusted for age (continuous), sex (binary), residence (categorical), education level (categorical), household income (categorical), smoking (binary), alcohol drinking (binary), physical activity (continuous), total energy intake (continuous), total fat intake (continuous), BMI (continuous), T2DM (binary), hypertension (binary).

Abbreviations: MMSE, mini-mental state examination; UCd, urinary cadmium; Ca, calcium; Fe, ferrum; Mg, magnesium; P, phosphorus; Se, selenium; BMI, body mass index; T2DM, type 2 diabetes mellitus.

Table S7. Results of the combined group regression model for urinary cadmium and serum minerals for subgroups of educational level.

Subgroups	Minerals	Exposure	Beta (95%CI)				$P_{\text{-interaction}}$
			High UCd and low minerals	Low UCd and low minerals	High UCd and high minerals	Low UCd and high minerals	
Junior high school or below	Ca	UCd	Reference	0.11 (0.03, 0.20)	0.07 (-0.01, 0.15)	0.21 (0.13, 0.30)	0.996
	Fe	UCd	Reference	0.15 (0.07, 0.24)	0.03 (-0.05, 0.11)	0.13 (0.04, 0.22)	0.996
	Mg	UCd	Reference	0.11 (0.02, 0.20)	0.06 (-0.02, 0.14)	0.20 (0.12, 0.29)	0.996
	P	UCd	Reference	0.12 (0.03, 0.20)	0.12 (0.04, 0.21)	0.24 (0.16, 0.33)	0.996
	Se	UCd	Reference	0.13 (0.05, 0.22)	0.16 (0.09, 0.24)	0.32 (0.23, 0.41)	0.745
Senior high school or above	Ca	UCd	Reference	0.03 (-0.05, 0.11)	0.01 (-0.07, 0.09)	0.05 (-0.02, 0.13)	0.867
	Fe	UCd	Reference	-0.02 (-0.10, 0.06)	-0.02 (-0.10, 0.06)	0.07 (-0.01, 0.15)	0.867
	Mg	UCd	Reference	0.03 (-0.05, 0.11)	0.08 (0.00, 0.16)	0.13 (0.05, 0.21)	0.867
	P	UCd	Reference	0.07 (-0.01, 0.15)	0.04 (-0.04, 0.12)	0.04 (-0.03, 0.12)	0.867
	Se	UCd	Reference	0.08 (-0.01, 0.16)	0.10 (0.03, 0.18)	0.12 (0.04, 0.21)	0.867

Models were adjusted for age (continuous), sex (binary), residence (categorical), education level (categorical), household income (categorical), smoking (binary), alcohol drinking (binary), physical activity (continuous), total energy intake (continuous), total fat intake (continuous), BMI (continuous), T2DM (binary), hypertension (binary), UCr (continuous). The $P_{\text{-interaction}}$ values were adjusted for multiple testing using the FDR method.

Abbreviations: MMSE, mini-mental state examination; UCd, urinary cadmium; Ca, calcium; Fe, ferrum; Mg, magnesium; P, phosphorus; Se, selenium; BMI, body mass index; T2DM, type 2 diabetes mellitus, UCr, urinary creatinine.

Table S8. Association of urinary cadmium and serum minerals with MMSE in overall population excluding all individuals with concentrations of urinary cadmium or minerals above the 99th percentile.

Exposure	Beta (95%CI)	Q1	Q2	Q3	Q4	*P*-trend
UCd	-0.027 (-0.050, -0.005)	Reference	0.00 (-0.06, 0.06)	-0.05 (-0.11, 0.01)	-0.06 (-0.13, 0.00)	0.024
Ca	0.036 (0.014, 0.058)	Reference	0.06 (0.00, 0.12)	0.09 (0.03, 0.15)	0.09 (0.03, 0.15)	0.003
Fe	0.018 (-0.005, 0.041)	Reference	0.06 (0.00, 0.12)	0.05 (-0.02, 0.11)	0.05 (-0.02, 0.11)	0.204
Mg	0.052 (0.030, 0.074)	Reference	0.12 (0.05, 0.18)	0.13 (0.07, 0.19)	0.14 (0.08, 0.21)	<0.001
P	0.040 (0.017, 0.064)	Reference	0.01 (-0.06, 0.07)	0.07 (0.00, 0.13)	0.11 (0.05, 0.18)	<0.001
Se	0.084 (0.062, 0.107)	Reference	0.13 (0.07, 0.20)	0.21 (0.15, 0.28)	0.21 (0.15, 0.27)	<0.001

Models were adjusted for age (continuous), sex (binary), residence (categorical), education level (categorical), household income (categorical), smoking (binary), alcohol drinking (binary), physical activity (continuous), total energy intake (continuous), total fat intake (continuous), BMI (continuous), T2DM (binary), hypertension (binary).

Abbreviations: MMSE, mini-mental state examination; UCd, urinary cadmium; Ca, calcium; Fe, ferrum; Mg, magnesium; P, phosphorus; Se, selenium; BMI, body mass index; T2DM, type 2 diabetes mellitus.

Table S9. Association of urinary cadmium and serum minerals with MMSE in overall population without multiple interpolation of covariates.

Exposure	Beta (95%CI)	Q1	Q2	Q3	Q4	*P*-trend
UCd	-0.019 (-0.049, 0.012)	Reference	-0.05 (-0.13, 0.03)	-0.06 (-0.14, 0.02)	-0.06 (-0.15, 0.03)	0.172
Ca	0.014 (-0.016, 0.043)	Reference	0.04 (-0.04, 0.12)	0.05 (-0.04, 0.13)	0.03 (-0.06, 0.11)	0.520
Fe	0.016 (-0.016, 0.048)	Reference	0.03 (-0.06, 0.13)	0.02 (-0.07, 0.11)	0.02 (-0.07, 0.11)	0.852
Mg	0.040 (0.012, 0.069)	Reference	0.13 (0.05, 0.22)	0.14 (0.06, 0.22)	0.13 (0.04, 0.21)	0.005
P	0.015 (-0.014, 0.044)	Reference	-0.04 (-0.11, 0.04)	0.00 (-0.09, 0.08)	0.06 (-0.03, 0.15)	0.276
Se	0.086 (0.054, 0.118)	Reference	0.17 (0.08, 0.26)	0.24 (0.15, 0.33)	0.24 (0.15, 0.33)	<0.001

Models were adjusted for age (continuous), sex (binary), residence (categorical), education level (categorical), household income (categorical), smoking (binary), alcohol drinking (binary), physical activity (continuous), total energy intake (continuous), total fat intake (continuous), BMI (continuous), T2DM (binary), hypertension (binary).

Abbreviations: MMSE, mini-mental state examination; UCd, urinary cadmium; Ca, calcium; Fe, ferrum; Mg, magnesium; P, phosphorus; Se, selenium; BMI, body mass index; T2DM, type 2 diabetes mellitus.

Table S10. Characteristics of participants included in CHNS 2015 excluding those with T2DM or hypertension.

	Total (n=3538)	Male (n=1542)	Female (n=1996)	*P* value
Age, year	55.08 (10.01)	55.79 (10.21)	54.54 (9.82)	<0.001
Rural, n (%)	1327 (37.5)	554 (35.9)	773 (38.7)	0.095
**Education, n (%)**				0.001
Primary school or below	2397 (67.8)	993 (64.4)	1404 (70.3)	
Junior high school	772 (21.8)	366 (23.7)	406 (20.3)	
Senior high school or above	369 (10.4)	183 (11.9)	186 (9.3)	
**Annual household income, yuan (%)**				0.773
Low	1381 (39.0)	592 (38.4)	789 (39.5)	
Medium	1235 (34.9)	548 (35.5)	687 (34.4)	
High	558 (15.8)	238 (15.4)	320 (16.0)	
Very high	364 (10.3)	164 (10.6)	200 (10.0)	
Smoker, n (%)	908 (25.7)	873 (56.6)	35 (1.8)	<0.001
Alcohol user, n (%)	954 (27.0)	822 (53.3)	132 (6.6)	<0.001
Physical activity, MET h/week	117.78 [52.85, 226.52]	130.00 [52.43, 250.65]	111.48 [52.88, 211.92]	0.013
Total energy intake, kcal	1935.28 [1537.48, 2419.58]	2123.30 [1702.69, 2653.11]	1796.39 [1424.84, 2222.98]	<0.001
Total fat intake, g	73.12 [51.85, 102.11]	80.25 [57.83, 110.85]	67.58 [47.89, 94.20]	<0.001
BMI, kg/m^2	23.42 [21.45, 25.66]	23.38 [21.33, 25.64]	23.43 [21.56, 25.67]	0.221
Urinary creatinine, μmol/L/24h	6004.00 [3371.75, 9604.25]	7097.00 [4046.25, 11053.25]	5269.00 [3016.75, 8273.75]	<0.001
MMSE score	27.59 (4.25)	27.83 (3.95)	27.41 (4.46)	0.003
Ca, mmol/L	2.41 (0.13)	2.42 (0.13)	2.41 (0.13)	0.293
Mg, mmol/L	0.91 (0.08)	0.91 (0.08)	0.90 (0.08)	<0.001
Fe, μmol/L	18.80 [14.60, 23.80]	20.90 [16.50, 26.40]	17.30 [13.50, 21.60]	<0.001
P, mmol/L	1.19 [1.07, 1.30]	1.12 [1.01, 1.24]	1.23 [1.13, 1.34]	<0.001

Se, μg/L	85.06 [71.58, 97.65]	85.06 [71.90, 98.20]	85.03 [71.36, 97.39]	0.574
UCd, μg/L	0.61 [0.29, 1.32]	0.67 [0.31, 1.38]	0.57 [0.28, 1.26]	0.003

Abbreviations: CHNS, China Health and Nutrition Survey; T2DM, type 2 diabetes mellitus; MET, metabolic equivalent; BMI, body mass index; MMSE, mini-mental state examination; Ca, calcium; Fe, ferrum; Mg, magnesium; P, phosphorus; Se, selenium; UCd, urinary cadmium.

Data were presented as the mean (SD), median [IQR], or frequency (percentage).

Table S11. Association of urinary cadmium and serum minerals with MMSE in overall population excluding patients with hypertension and type 2 diabetes mellitus.

Exposure	Beta (95%CI)	Q1	Q2	Q3	Q4	$P_{\text{-trend}}$
UCd	-0.041 (-0.072, -0.010)	Reference	0.02 (-0.07, 0.10)	-0.02 (-0.11, 0.07)	-0.09 (-0.18, -0.01)	0.025
Ca	0.061 (0.030, 0.091)	Reference	0.11 (0.03, 0.19)	0.12 (0.04, 0.21)	0.15 (0.06, 0.24)	<0.001
Fe	0.020 (-0.011, 0.051)	Reference	0.04 (-0.04, 0.13)	0.04 (-0.04, 0.13)	0.04 (-0.04, 0.13)	0.344
Mg	0.057 (0.027, 0.088)	Reference	0.13 (0.05, 0.22)	0.23 (0.15, 0.31)	0.15 (0.06, 0.24)	<0.001
P	0.038 (0.006, 0.070)	Reference	0.00 (-0.08, 0.09)	0.05 (-0.04, 0.14)	0.11 (0.02, 0.20)	0.011
Se	0.063 (0.032, 0.095)	Reference	0.10 (0.02, 0.19)	0.21 (0.12, 0.30)	0.16 (0.07, 0.24)	<0.001

Models were adjusted for age (continuous), sex (binary), residence (categorical), education level (categorical), household income (categorical), smoking (binary), alcohol drinking (binary), physical activity (continuous), total energy intake (continuous), total fat intake (continuous), BMI (continuous), T2DM (binary), hypertension (binary).

Abbreviations: MMSE, mini-mental state examination; UCd, urinary cadmium; Ca, calcium; Fe, ferrum; Mg, magnesium; P, phosphorus; Se, selenium; BMI, body mass index; T2DM, type 2 diabetes mellitus.

Table S12. Association of serum cadmium and minerals with MMSE in overall population.

Exposure	Beta (95%CI)	Q1	Q2	Q3	Q4	*P*-trend
SCd	-0.024 (-0.045, -0.003)	Reference	0.03 (-0.03, 0.09)	0.06 (0.00, 0.12)	-0.04 (-0.10, 0.02)	0.547
Ca	0.041 (0.020, 0.062)	Reference	0.06 (0.00, 0.11)	0.09 (0.03, 0.15)	0.09 (0.03, 0.15)	0.001
Fe	0.014 (-0.008, 0.036)	Reference	0.05 (-0.01, 0.11)	0.04 (-0.02, 0.10)	0.05 (-0.01, 0.11)	0.156
Mg	0.052 (0.031, 0.073)	Reference	0.09 (0.04, 0.15)	0.12 (0.06, 0.18)	0.14 (0.08, 0.20)	<0.001
P	0.038 (0.016, 0.061)	Reference	0.00 (-0.06, 0.06)	0.06 (0.00, 0.12)	0.11 (0.05, 0.17)	<0.001
Se	0.076 (0.055, 0.098)	Reference	0.13 (0.07, 0.18)	0.21 (0.15, 0.27)	0.19 (0.13, 0.25)	<0.001

Models were adjusted for age (continuous), sex (binary), residence (categorical), education level (categorical), household income (categorical), smoking (binary), alcohol drinking (binary), physical activity (continuous), total energy intake (continuous), total fat intake (continuous), BMI (continuous), T2DM (binary), hypertension (binary).

Abbreviations: MMSE, mini-mental state examination; SCd, serum cadmium; Ca, calcium; Fe, ferrum; Mg, magnesium; P, phosphorus; Se, selenium; BMI, body mass index; T2DM, type 2 diabetes mellitus.
